# Cranberry and Grape Seed Extracts Inhibit the Proliferative Phenotype of Oral Squamous Cell Carcinomas

**DOI:** 10.1093/ecam/nen047

**Published:** 2010-10-18

**Authors:** Kourt Chatelain, Spencer Phippen, Jonathan McCabe, Christopher A. Teeters, Susan O'Malley, Karl Kingsley

**Affiliations:** Department of Biomedical Sciences, School of Dental Medicine, University of Nevada, Las Vegas, USA

## Abstract

Proanthocyanidins, compounds highly concentrated in dietary fruits, such as cranberries and grapes, demonstrate significant cancer prevention potential against many types of cancer. The objective of this study was to evaluate cranberry and grape seed extracts to quantitate and compare their anti-proliferative effects on the most common type of oral cancer, oral squamous cell carcinoma. Using two well-characterized oral squamous cell carcinoma cell lines, CAL27 and SCC25, assays were performed to evaluate the effects of cranberry and grape seed extract on phenotypic behaviors of these oral cancers. The proliferation of both oral cancer cell lines was significantly inhibited by the administration of cranberry and grape seed extracts, in a dose-dependent manner. In addition, key regulators of apoptosis, caspase-2 and caspase-8, were concomitantly up-regulated by these treatments. However, cranberry and grape seed extracts elicited differential effects on cell adhesion, cell morphology, and cell cycle regulatory pathways. This study represents one of the first comparative investigations of cranberry and grape seed extracts and their anti-proliferative effects on oral cancers. Previous findings using purified proanthocyanidin from grape seed extract demonstrated more prominent growth inhibition, as well as apoptosis-inducing, properties on CAL27 cells. These observations provide evidence that cranberry and grape seed extracts not only inhibit oral cancer proliferation but also that the mechanism of this inhibition may function by triggering key apoptotic regulators in these cell lines. This information will be of benefit to researchers interested in elucidating which dietary components are central to mechanisms involved in the mediation of oral carcinogenesis and progression.

## 1. Introduction

A growing interest has developed in the fields of nutrition, dietetics and complementary medicine to identify dietary components and botanical or nutritional supplements for their specific chemopreventive and chemotherapeutic potentials [1, 2]. The consumption of specific foods or nutrients, such as fruits and vegetables, may be both a convenient and cost-effective method for the administration of beneficial and protective bioactive compounds. However, more detailed information is needed to identify the active components in these foods to evaluate dose-response relationships and toxicity, and to adjust for potential confounders in order to make these recommendations.

Multivariate meta-regression analysis from previous nutrition studies has revealed that the reduction in oral cancer risk by fruit consumption was significantly influenced by the type of fruit consumed, with other factors less significant [3]. This larger protective effect from developing oral cancer was most closely associated with citrus fruits and berries, rather than overall fruit consumption, even after adjusting for sex, age, tobacco or alcohol use. Based upon these studies and other epidemiologic and laboratory-based projects, a large number of anti-cancer agents derived from fruits and vegetables, particularly citrus fruits and berries, have been identified. These include flavonoids and polyphenolics, carotenoids, dithioltiones, glucosinolates, indoles, isothiocyanates, protease inhibitors, plant sterols, allium compounds, limonenes, selenium, vitamin C, vitamin E and dietary fiber [1, 4].

An interest has developed in one class of these compounds which exhibit chemopreventive and chemotherapeutic potential in many stages of oral carcinogenesis, the proanthocyanidins, which are highly concentrated in certain dietary fruits, nuts and berries. Proanthocyanidins (PACs) are polyphenolic compounds derived from common dietary foods such as grapes, cranberries and almonds, as well as chocolate and cacao beans [5–9]. Recent evidence suggests that PACs exhibit cytotoxicity against some cancers, including colon, breast and prostate cancers [10–15]. Moreover, studies involving raspberry-, grape- and grape seed-derived PACs have recently demonstrated selective inhibition of oral cancer phenotypes, particularly in oral squamous cell carcinomas (OSCC) [16, 17].

To more closely examine the potential relationship between PACs and the inhibition of oral cancer phenotypes, the specific effects of grape-seed extracts (GSE)-derived PAC administration on the *in vitro* proliferation of the most common of oral cancers, OSCC were assessed [18]. The results demonstrated that the administration of GSE-derived PACs was sufficient to reduce the proliferation of an OSCC cell line, CAL27, in a dose-dependent manner [18]. Moreover, the effects of GSE-PACs were more selective, and intensely specific, for the OSCC cell line compared with non-cancerous controls, suggesting a possible selective effect that may render oral cancers more susceptible to the apoptosis-inducing and proliferation-inhibiting effects of PACs.

Although the aforementioned studies have provided crucial information towards our understanding of oral cancer growth inhibition *in vitro*, thus far none have compared various PAC-containing extracts for their ability to inhibit oral cancer growth. Studies which demonstrated the inhibition of oral cancer proliferation *in vitro* using flavonoid- and proanthocyanidin-rich extracts from grape seed- [16], raspberry- [17] and cranberry-extracts [9] suggest that PACs derived from many dietary sources may function in oral cancer chemoprevention. No published study to date, however, has incorporated multiple PACs to provide comparative evaluation of these anti-proliferative and growth-inhibitory properties in multiple oral cancer cell lines.

Assessing dietary and nutrition information to provide specific recommendations for patients with increased risk for developing oral cancers is extremely challenging, partly due to the difficulties surrounding the confounding effects of other behaviors and risks, but also due to the dearth of information regarding the specific effects of particular dietary components on oral cancer phenotypes. Therefore, the aim of this study was to evaluate two PAC sources, cranberry extract (CE) and GSE, to investigate and compare their anti-proliferative and growth-inhibitory effects. More importantly, these results can be contextualized with our previous studies of purified GSE-derived PACs, to more accurately assess the potential value for further studies of PAC-induced inhibition of oral cancers and for specific recommendations for testing of PACs for future animal and human clinical trials.

## 2. Methods

### 2.1. Materials

Grape seed extract (GSE) (Lot #0549BG1980) from US wine grapes (*Vitus vinifera*) and cranberry (*Vaccinium macrocarpon*) extract (CE) (Lot #1367CG1936) were obtained from GNC Prevention Nutrition^®^ (Pittsburgh, PA). CE contains oligomeric and polymeric polyphenols, including PACs [9], while GSE has been demonstrated to contain 95% PACs, with 80–90% oligomeric PACs, including dimers, trimers, tetramers, and a small amount of monomeric flavonoids [19, 20].

### 2.2. Cell Culture

The human OSCC cell lines used in this study, CAL27 and SCC25, were obtained from American Type Culture Collection (ATCC: Manassas, VA). CAL27 cells were maintained in Dulbecco's Modified Eagle's Medium (DMEM) with 4 mM l-glutamine, adjusted to contain 3.7 g/l sodium bicarbonate and 4.5 g/l glucose from Hyclone (Logan, UT). SCC25 cells were maintained in a 1 : 1 mixture of DMEM and Ham's F12 medium with 2.5 mM l-glutamine, modified to contain 15 mM HEPES, 0.5 mM sodium pyruvate, and 1.2 g/l sodium bicarbonate (ATCC), supplemented with 400 ng/ml hydrocortisone from Sigma-Aldrich (St Louis, MO). Media for all cell lines was supplemented with 10% fetal bovine serum (FBS) and 1% Penicillin (10 000 units/ml)–Streptomycin (10 000 *μ*g/ml) solution (HyClone). Cell cultures were maintained in 75 cm^2^ BD Falcon tissue-culture treated flasks (Bedford, MA) at 37°C and 5% CO_2_ in humidified chambers.

### 2.3. Proliferation

Proliferation assays of CAL27 and SCC25 cells were performed in the appropriate complete media, with and without the addition of GSE and CE. The total concentrations of GSE and CE used were between 10 and 80 *μ*g/ml, added to the complete media prior to the start of each experimental assay and cell plating. Four independent replications of each experiment (CE, GSE) for each cell line (CAL27, SCC25) were performed (*n* = 288), each consisting of eight wells per experimental concentration (0, 10, 20, 30, 40, 50, 60, 70 and 80 *μ*g/ml, *n* = 72 wells/plate). Cells were plated in Corning Costar high-throughput, 96-well assay plates (Corning, NY) at a concentration of 1.2 × 10^4^ cells per well, which roughly approximates 30–40% confluence per well at the onset of each assay; proliferation was subsequently measured over 3 days. Cultured cells were fixed after 24 h (day 1), 48 h (day 2) and 72 h (day 3) using 50 *μ*l of 10% buffered formalin, and were stained with crystal violet 1% aqueous solution (Fisher Scientific: Fair Lawn, NJ). The relative absorbance was measured at 630 nm using a Bio-Tek ELx808 microplate reader (Winooski, VT). Data were analyzed and graphed using Microsoft Excel (Redmond, WA) and SPSS (Chicago, IL). Four separate, independent replications of each experimental condition were performed (CE, GSE) for each cell line (CAL27, SCC25) (*n* = 288).

### 2.4. Adhesion

Cell adhesion assays of CAL27 and SCC25 cells were performed, as previously described [21, 22], in uncoated Corning Costar 96-well assay plates at a concentration of 1.2 × 10^5^ cells per well (100 *μ*l of 1.2 × 10^6^ cells/ml solution). Cells were suspended in the appropriate media, as described above in cell culture methods; with no additives, with and without the addition of GSE and CE at the indicated concentrations. Cells were allowed to attach for 30 min at 37°C with one modification to the standard adhesion assay protocol. This modification eliminated the plate suspension step, in which non-adherent cells are generally removed by suspending the plate upside-down in a rotating tank of PBS, as previously described [18, 23]. Following the incubation period, cells were fixed using 50 *μ*l of 10% buffered formalin and were subsequently stained with crystal violet 1% aqueous solution. The relative absorbance was then measured at 630 nm using a Bio-Tek ELx808 microplate reader. Data were analyzed and graphed using Microsoft Excel. Three separate, independent replications of each experimental condition were performed (CE, GSE) for each cell line (CAL27, SCC25) (*n* = 216).

### 2.5. Statistics

The differences between treatments were measured using a *t* distribution, *α* =  .05. All samples were analyzed using two-tailed *t*-tests as departure from normality can make more of a difference in a one-tailed than in a two-tailed *t*-test. As long as the sample size is even moderate (>20) for each group, quite severe departures from normality make little practical difference in the conclusions reached from these analyses [24]. However, analyses involving multiple two sample *t*-tests may have a higher probability of Type I error, leading to false rejection of the null hypothesis, H_0_ [24]. To confirm the effects observed from these experiments and minimize the possibility of Type I error, further analysis of the data was facilitated with ANOVA using SPSS (Chicago, IL) to more accurately assess the relationship and statistical significance among and between groups.

### 2.6. Microscopy of Cell Morphology and Viability

Cells were visualized with a Zeiss Axiovert 40 inverted microscope (Gottingen, Germany), and images were captured at 200 × magnification with a Canon PowerShot G6 digital camera (Tokyo, Japan). Digital images were subsequently processed using Adobe Photoshop (San Jose, CA) Image Analysis tools. In brief, several wells of CAL27 and SCC25 cells were photographed from the adhesion assays and also from each time point of the proliferation assays (day 1, day 2, day 3) at each concentration of the treatment conditions (CE, GSE; 0–80 *μ*g/ml) to visualize any effects on cell morphology, pre-fixation. In addition, several wells of cells were also fixed at these time points using 50 *μ*l of 10% buffered formalin and subsequently stained using crystal violet 1% aqueous solution to quantitatively document cell morphology, percent of cell spreading and confluence. Additionally, at each time point, several wells were stained using Trypan Blue, and live cells were enumerated to determine viability, as previously described [25, 26].

### 2.7. RT-PCR

RNA was isolated from 1.5 × 10^7^ cells of CAL27 and SCC25 cells at 24 h after CE or GSE administration using ABgene Total RNA Isolation Reagent (Epsom, Surrey, UK) and the procedure recommended by the manufacturer. RT-PCR was performed on total RNA with the ABgene Reverse-iT One-Step RT-PCR Kit (ReadyMix Version) and a Mastercycler gradient thermocycler (Eppendorf: Hamburg, Germany). The following primers for p53 [27], c-myc [28], ornithine decarboxylase (ODC) [29], caspase-2 [30], caspase-3 [30], caspase-8 [31], bax [30] and glyceraldehyde-3-phosphate dehydrogenase GAPDH [29], synthesized by SeqWright (Houston, TX), were used:

 p53 forward primer, ACCAGGGCAGCTACGGTTTC;
 p53 reverse primer, CCTGGGCATCCTTGAGTTCC;
 c-myc forward primer, TCCAGCTTGTACCTGCAGGATCTGA;
 c-myc reverse primer, CCTCCAGCAGAAGGTGATCCAGACT;
 ODC forward primer, AATCAACCCAGCGTTGGACAA;
 ODC reverse primer, ACATCACATAGTAGATCGTCG;
 caspase-2 forward primer, TGGCATATAGGTTGCAGTCTCGG;
 caspase-2 reverse primer, TGTTCTGTAGGCTTGGGCAGTTG;
 caspase-3 forward primer, ACATGGAAGCGAATCAATGGACTC;
 caspase-3 reverse primer, AAGGACTCAAATTCTGTTGCCACC;
 caspase-8 forward primer, GATATTGGGGAACAACTGGAC;
 caspase-8 reverse primer, CATGTCATCATCCAGTTTGCA;
 bax forward primer, GGTTTCATCCAGGATCGAGACGG;
 bax reverse primer, ACAAAGATGGTCACGGTCTGCC;
 GAPDH forward primer, ATCTTCCAGGAGCGAGATCC;
 GAPDH reverse primer, ACCACTGACACGTTGGCAGT.

One microgram of template (total) RNA was used for each reaction. The reverse transcription step ran for 30 min at 47°C, followed by denaturation for 2 min at 94°C. Thirty-five amplification cycles were run, consisting of 20 s denaturation at 94°C, 30 s of annealing at 58°C, and 6.5 min of extension at 72°C. Final extension was run for 5 min at 72°C. Reaction products were separated by gel electrophoresis using Reliant 4% NuSieve^®^ 3 : 1 Plus Agarose gels (Lonza: Rockland, ME). Bands were visualized by UV illumination of ethidium-bromide-stained gels and captured using a Kodak Gel Logic 100 Imaging System and 1D Image Analysis Software (Eastman Kodak: Rochester, NY). Quantitation of RT-PCR band densitometry was performing using Adobe (San Jose, CA) Photoshop imaging software, Image Analysis tools.

## 3. Results

### 3.1. Proliferation

#### 3.1.1. CAL27

CAL27 cells were grown in 96-well assay plates and their proliferation was measured over 3 days in four separate, independent experimental trials to determine if the administration of either CE or GSE was sufficient to inhibit cellular proliferation. The results of these experiments demonstrated that CAL27 cellular proliferation was inhibited in a dose-dependent manner by both CE and GSE, although their effects were distinct from one another. The maximal growth inhibitory concentrations (GI_MAX_) of CE and GSE on CAL27 cells also exhibited distinctive patterns.

**Figure 1:**
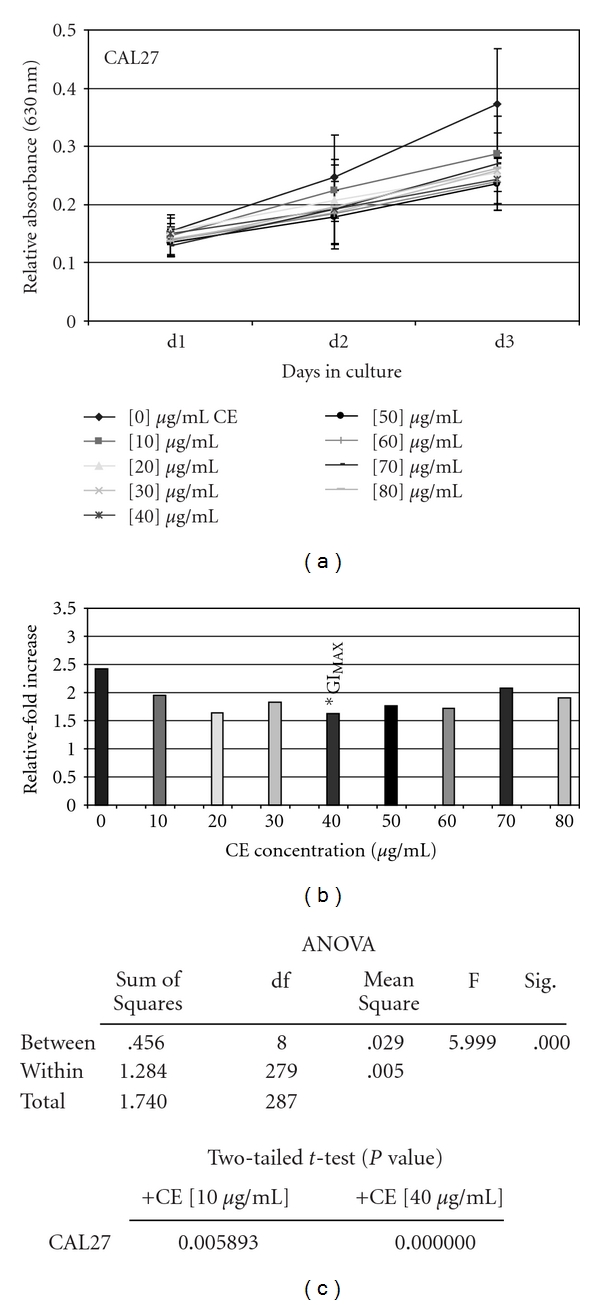
CE administration significantly inhibited CAL27 proliferation *in vitro*. CAL27 cells were plated in 96-well assay plates with media containing 10% fetal bovine serum (FBS) in the absence and presence of increasing CE concentrations (0–80 *μ*g/ml) and were allowed to proliferate for 3 days. The addition of CE induced dose-dependent inhibition of proliferation up to GI_MAX_ (–34%) at 40 *μ*g/ml (a) (*n* = 288, *P* <  .01). Relative-fold increase in proliferation confirmed GI_MAX_ at 40 *μ*g/ml (b), graphed as the relative fold proliferation—measured by day 3 measurement average minus day 1 measurement average (d3–d1). Two-tailed *t*-test and one-way ANOVA confirm statistical significance of CE-induced proliferation inhibition of CAL27 at all concentrations (c). A colour version of this figure is available online as supplementary data.

All concentrations of CE (10–80 *μ*g/ml) were sufficient to inhibit proliferation of CAL27 cells (Figure 1), which exhibited dose-dependent relationships (Figure 1(a)). To reduce the proliferation-stimulating effects of trypsin and plating of cells into each experimental assay, previously observed between day 0 and day 1 in many proliferation assays [18, 32], the relative change in proliferation was measured between day 3 and day 1. This analysis revealed the GI_MAX_ for CE on CAL27 cells was 40 *μ*g/ml, inhibiting CAL27 growth by 34% compared with the baseline treatment controls (Figure 1(b)); confirmation of the observations based directly from raw data (Figure 1(a)).

Two tailed *t*-tests, performed to validate the reduction in CAL27 proliferation at all CE concentrations, revealed that all experimental concentrations resulted in statistically significant inhibition of proliferation (Figure 1(c)) (*n* = 288, *P* <  .01). Because these analyses involved multiple two sample *t*-tests, with a higher probability of Type I error, ANOVA was performed to more accurately assess the relationship among groups. This analysis verified statistically significant differences between the experimental groups, but not within the groups (Figure 1(c)), further corroborating that statistically significant differences in CAL27 proliferation were induced with CE administration.

**Figure 2:**
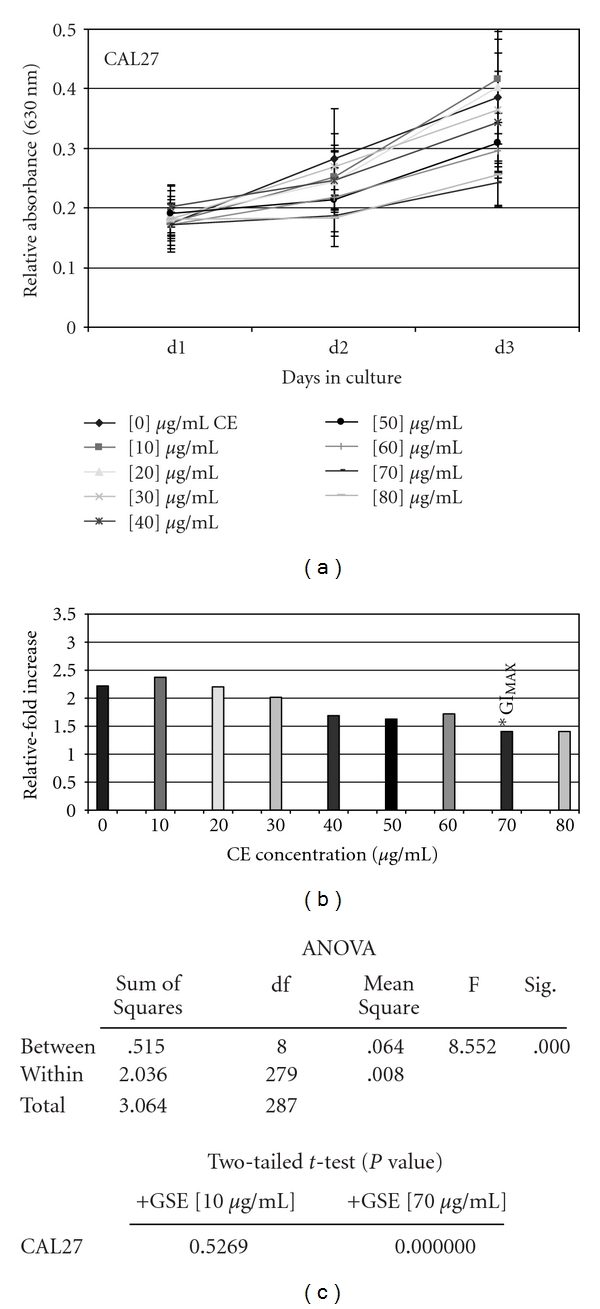
GSE administration inhibited CAL27 proliferation *in vitro*. CAL27 cells were plated in 96-well assay plates with media containing 10% fetal bovine serum (FBS) in the absence and presence of increasing GSE concentrations (0–80 *μ*g/ml) and were allowed to proliferate for 3 days. The addition of GSE at low concentration (10–20 *μ*g/ml) stimulated proliferation of CAL27 cells, while increasing concentrations elicited dose-dependent inhibition up to GI_MAX_ (–38%) at 70 *μ*g/ml (a) (*n* = 288, *P* <  .01). Relative-fold increase in proliferation confirmed GI_MAX_ at 70 *μ*g/ml (b), graphed as the relative fold proliferation—measured by day 3 measurement average minus day 1 measurement average (d3–d1). Two-tailed *t*-test and one-way ANOVA confirm statistical significance of GSE proliferation inhibition of CAL27 at concentrations >50 *μ*g/ml (c). A colour version of this figure is available online as supplementary data.

The administration of GSE (10–20 *μ*g/ml), however, did not inhibit proliferation of CAL27 cells, but rather stimulated proliferation slightly—although this was not statistically significant (Figure 2). Higher concentrations of GSE were, however, sufficient to inhibit proliferation of CAL27 cells, exhibiting a strong dose-dependent relationship at concentrations >30 *μ*g/ml (Figure 2(a)). The analysis of relative change in proliferation between day 3 and day 1 revealed that the GI_MAX_ for GSE on CAL27 cells was 70 *μ*g/ml, inhibiting CAL27 proliferation by 38% compared with the baseline treatment controls (Figure 2(b)).

Two tailed *t*-tests, performed to validate each reduction in CAL27 proliferation at all GSE concentrations, revealed that only experimental concentrations above 50 *μ*g/ml represented statistically significant inhibitions in proliferation (Figure 2(c)). ANOVA confirmed statistically significant differences between the experimental groups, but not within the groups (Figure 1(c)), providing further validation of the statistical differences observed with GSE-induced inhibition of CAL27 proliferation.

#### 3.1.2. SCC25

SCC25 cells were also seeded in 96-well assay plates and their proliferation was measured over 3 days in four separate, independent experimental trials to determine if the administration of CE or GSE was sufficient to inhibit cellular proliferation. The results of these experiments demonstrated that SCC25 cellular proliferation was inhibited in a dose-dependent manner by both CE and GSE, although the inhibitory effects of these extracts were also distinct from one another. Unlike CAL27, however, the GI_MAX_ concentrations for both CE and GSE on the SCC25 cell line were identical.

**Figure 3:**
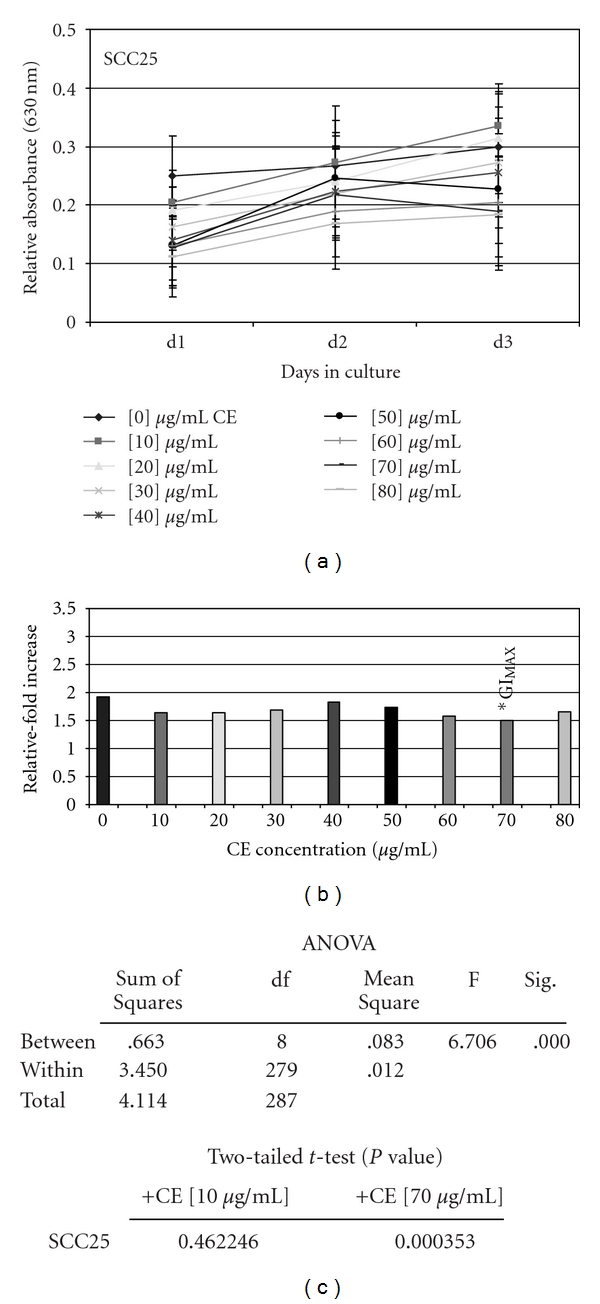
CE administration inhibited SCC25 proliferation *in vitro*. SCC25 cells were plated in 96-well assay plates with media containing 10% fetal bovine serum (FBS) in the absence and presence of increasing CE concentrations (0–80 *μ*g/ml) and were allowed to proliferate for 3 days. The addition of CE at low concentration (10–20 *μ*g/ml) stimulated proliferation of SCC25 cells, while increasing concentrations elicited dose-dependent inhibition up to GI_MAX_ (–36%) at 70 *μ*g/ml (a) (*n* = 288, *P* <  .01). Relative-fold increase in proliferation confirmed GI_MAX_ at 70 *μ*g/ml (b), graphed as the relative fold proliferation—measured by day 3 measurement average minus day 1 measurement average (d3–d1). Two-tailed *t*-test and one-way ANOVA confirm statistical significance of CE proliferation inhibition of SCC25 at concentrations >50 *μ*g/ml (c). A colour version of this figure is available online as supplementary data.

Low concentrations of CE (10–20 *μ*g/ml) did not inhibit proliferation of SCC25 cells, but rather stimulated proliferation slightly more than control cells without treatment—although this was not statistically significant (Figure 3). Higher concentrations of CE were, however, sufficient to inhibit proliferation of SCC25 cells, exhibiting a graduated, increasing dose-dependent relationship for inhibition (Figure 3(a)). The analysis of relative change in proliferation between day 3 and day 1 revealed that the GI_MAX_ for CE on SCC25 cells was 70 *μ*g/ml, demonstrating reduced proliferation of SCC25 by 36.3% compared with the baseline treatment controls (Figure 3(b)).

Two tailed *t*-tests, performed to validate each reduction in SCC25 proliferation at all CE concentrations, revealed that only experimental concentrations above 50 *μ*g/ml represented statistically significant inhibitions in proliferation (Figure 3(c)). ANOVA was performed to more accurately assess the relationship among groups. These results demonstrated statistically significant differences between the experimental groups, but not within the groups (Figure 3(c)), validating the statistical differences observed with CE-induced inhibition of SCC25 proliferation above 50 *μ*g/ml.

**Figure 4:**
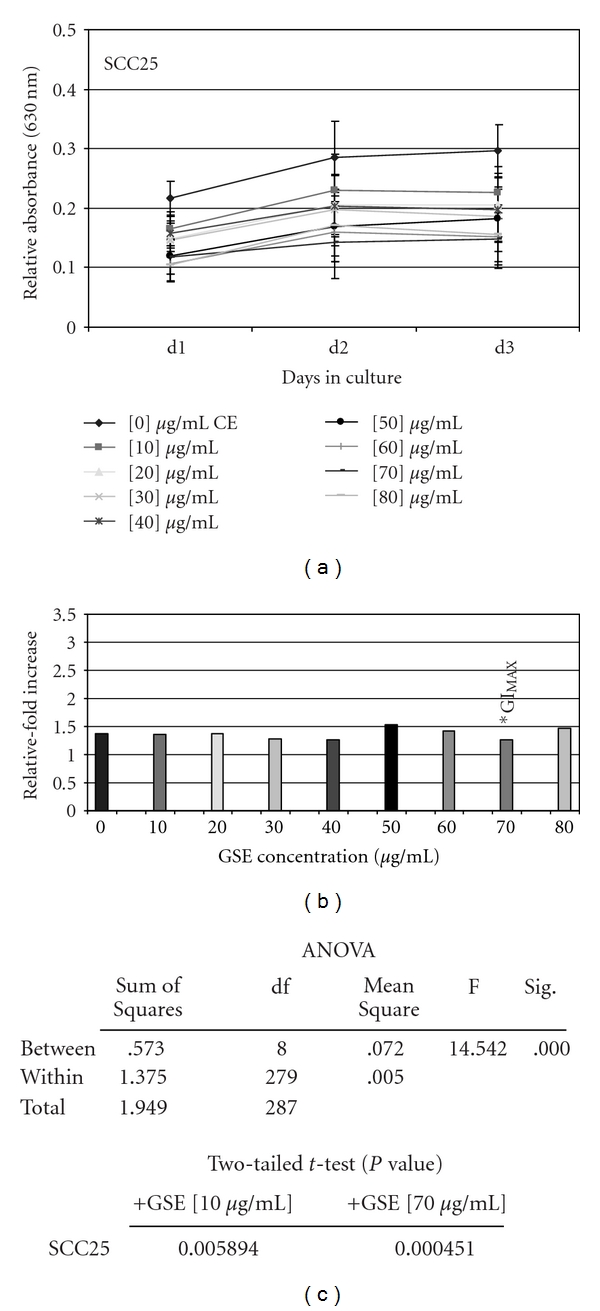
GSE administration significantly inhibited SCC25 proliferation *in vitro*. SCC25 cells were plated in 96-well assay plates with media containing 10% fetal bovine serum (FBS) in the absence and presence of increasing GSE concentrations (0–80 *μ*g/ml) and were allowed to proliferate for 3 days. The addition of GSE induced significant, dose-dependent inhibition of proliferation up to GI_MAX_ (–51%) at 70 *μ*g/ml (a) (*n* = 288, *P* <  .01). Relative-fold increase in proliferation confirmed GI_MAX_ at 70 *μ*g/ml (b), graphed as the relative fold proliferation—measured by day 3 measurement average minus day 1 measurement average (d3–d1). Two-tailed *t*-test and one-way ANOVA confirm statistical significance of GSE-induced proliferation inhibition of SCC25 at all concentrations (c). A colour version of this figure is available online as supplementary data.

All concentrations of GSE (10–80 *μ*g/ml) were sufficient to inhibit proliferation of SCC25 cells (Figure 4), exhibiting similar levels of proliferation inhibition that was dose-dependent (Figure 4(a)). The relative change in proliferation between day 3 and day 1 revealed that the GI_MAX_ for GSE on SCC25 cells was 70 *μ*g/ml, representing an inhibition of SCC25 growth by 51.2% compared with baseline treatment controls (Figure 4(b)).

Two tailed *t*-tests, performed to validate each reduction in SCC25 proliferation at all GSE concentrations, revealed that all experimental concentrations represented statistically significant inhibitions in proliferation (Figure 4(c)). ANOVA confirmed statistically significant differences between all of the experimental groups, but not within the groups (Figure 4(c)), providing further validation of the statistical differences observed with GSE-induced inhibition of SCC25 proliferation.

### 3.2. Adhesion

**Figure 5:**
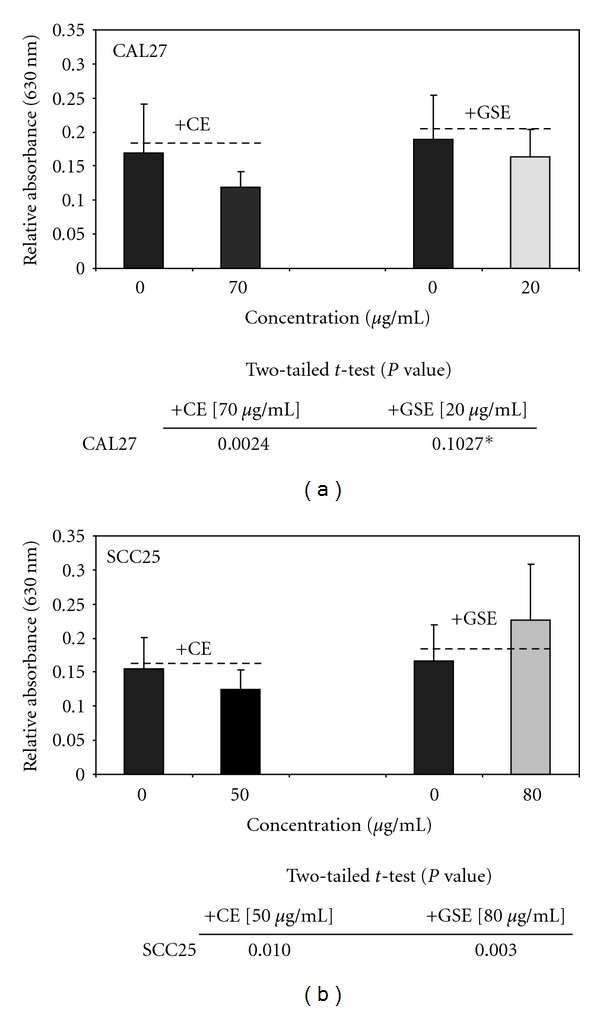
CE inhibited adhesion of OSCC cell lines *in vitro* while GSE had variable effects. The addition of CE reduced CAL27 (a) and SCC25 (b) adhesion significantly at 70 (–30%, *n* = 216, *P* <  .01) and 50 *μ*g/ml (–19%, *n* = 216, *P* =  .01), respectively. The addition of GSE, however, reduced CAL27 adhesion (not significant, *n* = 216, *P* =  .10) while significantly increasing SCC25 adhesion (+36.6%, *n* = 216, *P* <  .01). CE, GSE *P*-values and relative change percentages are presented in Table 1. A colour version of this figure is available online as supplementary data.

Based upon the results from the cellular proliferation assays, *in vitro* cellular adhesion assays were then performed using 30-min adhesion assays to determine if any of the proliferation-inhibitory responses to CE or GSE in these cell lines were correlated with quantifiable differences in adhesion (Table 1). The results of these assays revealed that CAL27 adhesion *in vitro* was altered by the administration of either CE or GSE. More specifically, baseline CAL27 cellular adhesion was reduced in a dose-dependent manner by increasing concentrations of CE, which were significant at all concentrations >50 *μ*g/ml (*n* = 120, *P* <  .01) (Figure 5); with the greatest effect observed at 70 *μ*g/ml (Figure 5(a)). The administration of GSE induced an almost immediate reduction in CAL27 adhesion, observed at a concentration of 20 *μ*g/ml, while further increasing concentrations exhibited diminishing inhibitory effects on adhesion; none were determined to be statistically significant (*n* = 216, *P* >  .05) (Figure 5(a)).

The administration of CE and GSE, however, exhibited dissimilar and less robust effects compared with the baseline levels of SCC25 cellular adhesion (Table 1). The administration of increasing concentrations of CE reduced SCC25 adhesion in a dose-dependent manner with maximal reduction in adhesion observed at 50 *μ*g/ml (*n* = 24, *P* <  .01), the only concentration representing a statistically significant reduction, with all higher concentrations exhibiting diminishing capacity to reduce SCC25 adhesion which were not significant (*n* = 192, *P* >  .05) (Figure 5(b)). The administration of increasing concentrations of GSE, however, induced measurable, though not significant, increases in SCC25 adhesion (*n* = 192, *P* >  .05), except with the maximal increase observed at 80 *μ*g/ml (*n* = 24, *P* <  .01) (Figure 5(b)).

### 3.3. Microscopy of Cell Morphology and Viability

**Figure 6:**
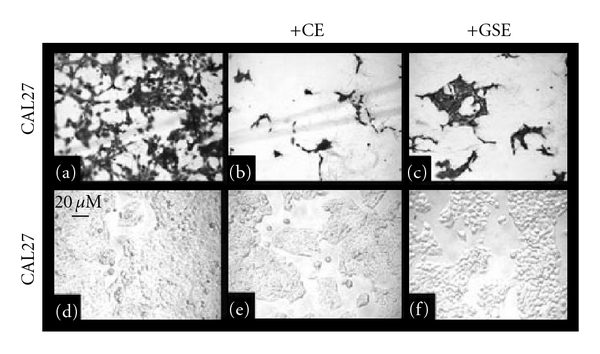
Effects of CE and GSE on CAL27 cell morphology *in vitro*. CAL27 cell morphology from proliferation assays, fixed with formalin and stained with crystal violet (a–c) or pre-fixation (d–f) demonstrate reduced proliferation and lower cell numbers under CE (b, e) or GSE (c, f) treatment compared with untreated controls (a, d). A colour version of this figure is available online as supplementary data.

**Figure 7:**
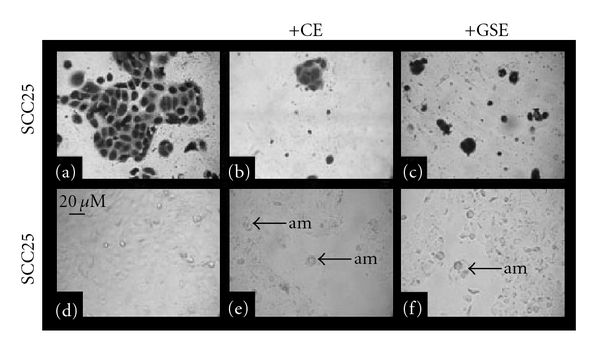
Effects of CE and GSE on SCC25 cell morphology *in vitro*. SCC25 cell morphology from proliferation assays, fixed with formalin and stained with crystal violet ((a–c)) or pre-fixation ((d–f)) demonstrate reduced proliferation and lower cell numbers under CE ((b, e)) or GSE ((c, f)) treatment compared with untreated controls ((a, d)). Some cells displayed altered morphology (am), which was not seen with either experimental treatment of CAL27 cells. A colour version of this figure is available online as supplementary data.

GI_MAX_ concentrations of CE and GSE were sufficient to inhibit proliferation of both cell lines, as well as inducing alterations to cellular adhesion; therefore, microscopy was performed to more accurately assess the qualitative effects of CE and GSE administration on CAL27 (Figure 6) and SCC25 cells (Figure 7). Compared with CAL27 controls without treatment (Figures 6(a) and 6(d)), the administration of CE at any concentration resulted in proliferation inhibition and reduced cell number, an observation clearly visible in all stained (Figure 6(b)) and unstained wells (Figure 6(e)). The administration of GSE also inhibited CAL27 proliferation at concentrations higher than 20 *μ*g/ml, and this reduction in proliferation was visible and apparent in stained (Figure 6(c)) and unstained wells (Figure 6(f)). Although cell proliferation was inhibited in both of the experimental groups (CE, GSE), significant alterations to cell morphology and cell spreading were not observed with either treatment or at any concentration.

The administration of CE and GSE on SCC25 cells was sufficient to inhibit growth and proliferation, but was also sufficient to induce alterations to cellular morphology (Figure 7). In comparison to SCC25 control cells without treatment (Figures 7(a) and 7(d)) the administration of CE at concentrations >50 *μ*g/ml significantly inhibited proliferation and induced phenotypic changes to cell morphology (Figures 7(b) and 7(e)). All concentrations of GSE were sufficient to significantly inhibit SCC25 proliferation and also induced visible phenotypic changes to cell morphology (Figures 7(c) and 7(f)).

**Figure 8:**
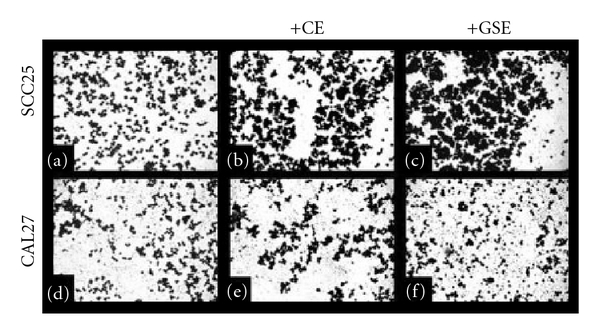
Effects of CE and GSE on cell morphology in 30-min adhesion assays. SCC25 ((a–c)) and CAL27 ((d–f)) cells from adhesion assays were fixed with formalin and stained with crystal violet. Analysis of captured images revealed that SCC25 cell morphology (a) was significantly altered and cell clustering (cc) increased by the administration of CE (b) and GSE (c) at GI_MAX_ concentrations (50, 80 *μ*g/ml, resp.) where cells were present; although significant areas of each experimental well (CE, GSE) had few, if any, adherent cells. CAL27 cells (d) exhibited no notable differences in cell clustering or cell–cell adhesion under CE (e) or GSE (f) administration. A colour version of this figure is available online as supplementary data.

Because these experimental groups (CE, GSE) induced differential responses and alterations to cell morphology in the CAL27 and SCC25 cell lines over the course of 3 days, additional microscopy was performed to determine if these effects could be observed in experiments with a shorter temporal component, such as the 30-min adhesion assays (Figure 8). SCC25 control cells (Figure 8(a)) revealed a marked increase in cell clustering and cell-cell adhesion with the administration of CE (Figure 8(b)) or GSE (Figure 8(c)). No notable differences in cell morphology or arrangement were noted, however, between CAL27 control cells (Figure 8(d)) and CE-treated (Figure 8(e)) or GSE-treated (Figure 8(f)) cells.

**Figure 9:**
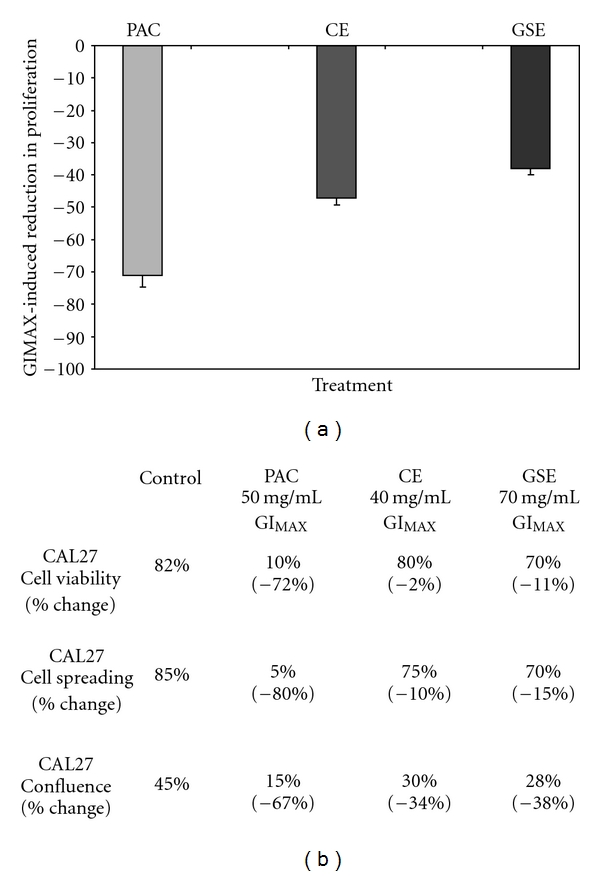
Proliferation inhibition of CAL27 comparison: PAC, CE and GSE. Previous results of proliferation inhibition of CAL27 with PAC were compared with the results of the current investigation. GI_MAX_ concentration of purified GSE-derived PAC (50 *μ*g/ml) exhibited a slightly more robust inhibition of CAL27 growth than CE or GSE (a). Comparison of current results with previous observations using purified GSE-derived PAC demonstrated that although all treatments (PAC, CE, GSE) reduced cell number and inhibited proliferation, to varying degrees, purified GSE-derived PAC most decreased cell viability (–72%) and cell spreading (–80%) (b). A colour version of this figure is available online as supplementary data.

Our previous work with CAL27 and purified GSE-derived PAC revealed that GI_MAX_ concentrations (50 *μ*g/ml) were sufficient not only to inhibit proliferation, but also to induce phenotypic changes in cell viability and morphology [18]. A comparison of the previous observations with this current study reveals notable differences in cell viability and cell spreading among treatment groups (Figure 9). For example, although CE and GSE reduced CAL27 proliferation (–34%, –38%, resp.), this effect was comparatively smaller than the effect of purified GSE-derived PAC (Figure 9(a)). Moreover, although cell number and confluence were reduced significantly by CE and GSE, cell viability and cell spreading were not—in stark contrast to the observations of CAL27 under the administration of purified GSE-derived PAC (Figure 9(b)).

### 3.4. RT-PCR

**Figure 10:**
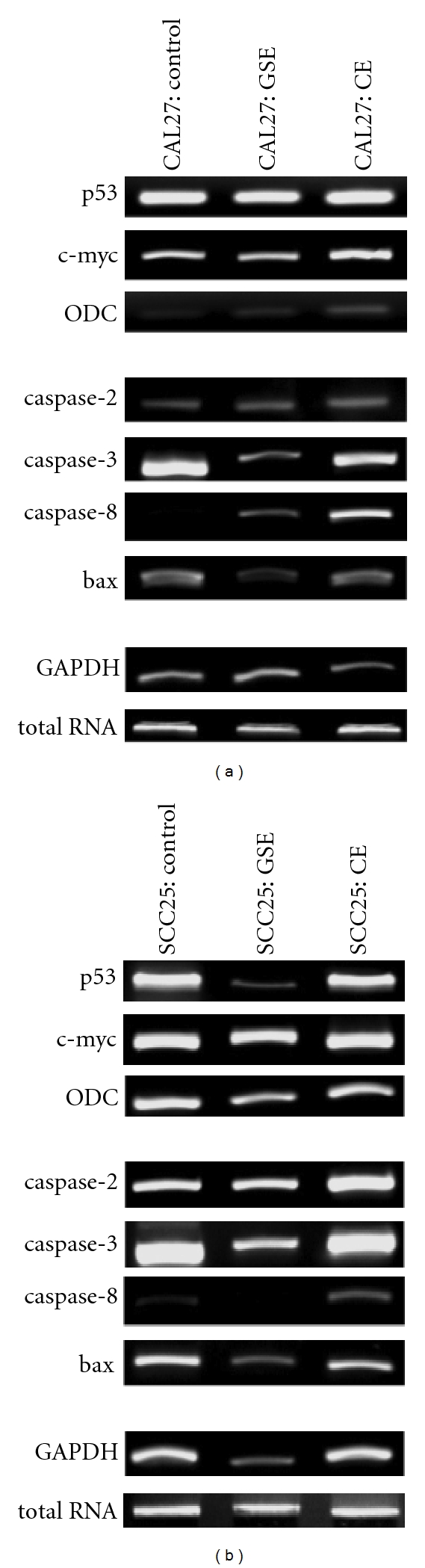
Total RNA and mRNA analysis. RT-PCR was performed on total RNA extracted from CAL27 (a) and SCC25 (b) cells at 24 h after CE or GSE administration; no significant changes were detected at 4 h (data not shown). Relative endpoint (RE) RT-PCR revealed that CE and GSE treatment increased expression of apoptosis-related mRNA (caspase-2, caspase-8) in both cell lines, which is first detectable at 24 h. CE significantly enhanced CAL27 expression of cell-cycle genes (p53, c-myc, ODC), while GSE reduced SCC25 expression of these same targets at this time point.

To determine if the differential effects of CE and GSE administration on these cell lines was, in part, due to differential expression of cell-cycle and apoptosis regulatory gene expression, RT-PCR was performed on total RNA isolated from cultured CAL27 and SCC25 cells (Figure 10). Using oligonucleotide primers specific for mRNA of the cell-cycle regulatory genes *p53*, *c-myc* and *ODC*, as well as the apoptosis regulatory genes *caspase-2, -3, -8* and *bax*, densitometric measurements of relative endpoint (RE) RT-PCR band intensity for mRNA expression of these genes were compared to endogenous expression from untreated CAL27 (Figure 10(a)) and SCC25 (Figure 10(b)) cells.

These analyses revealed that administration of GSE on CAL27 cells had no significant effect on the cell-cycle gene products tested, while CE administration induced an increase in expression of each: p53 (+44%), c-myc (+29%) and ODC (+371%) without significantly altering levels of total RNA (Figure 10(a)). Conversely, the administration of CE on SCC25 cells had no significant effect on cell-cycle gene products, while GSE administration reduced expression of each: p53 (−51%), c-myc (−7%) and ODC (−21%) without significantly altering total RNA (Figure 10(b)). GSE and CE treatment of CAL27 cells induced striking effects to mRNA levels of apoptosis-associated pathway modulators, increasing expression of caspase-2 by +239% and +327%, respectively and caspase-8 by +21 and +181%, respectively. GSE and CE treatment of SCC25 cells also induced increased expression of caspase-2 (+42%, +27%, resp.) and caspase-8 (+2%, +27%, respectively), although these increases were less robust. Interestingly, the administration of GSE was sufficient to reduce expression of bax and caspase-3 mRNA in both cell lines.

## 4. Discussion

Although the health benefits and disease fighting potential of proper diet and good nutrition are well established in the literature, more recent findings suggest that particular dietary components, such as fruits and vegetables, are significant protective factors against oral cancer [3]. Moreover, the overall level of protection and risk reduction associated with consumption of specific fruit and vegetables has suggested this protection may be more strongly correlated with particular citrus fruits and berries [16, 17]. These observations have provided strong evidence for the continued study of one group of compounds highly concentrated in these foods [5–9], proanthocyanidins (PACs), which demonstrated significant chemoprevention and chemotherapeutic potential against a wide range of cancers and more recently against oral cancers [16–18].

Although other studies have demonstrated that particular PAC-containing fractions of grape-seed, cranberry or raspberry extracts may exhibit cytotoxic or anti-proliferative effects, none have yet provided direct comparisons of the effects of PACs from differing sources in oral tumor cell lines. The overall goal of this study, therefore, was to evaluate two commercially available PAC sources, cranberry extract (CE) and grape seed extract (GSE), to investigate and compare their anti-proliferative and growth-inhibitory effects on well-characterized oral cancer cell lines *in vitro*. The initial working hypothesis was that both CE and GSE would be sufficient to mediate the proliferative phenotype of oral cancers, allowing for quantitative differences to be identified.

These analyses did confirm the inhibition of cellular proliferation observed in previous studies [16–18]. Notably, CE and GSE treatments significantly reduced cell growth and proliferation of both oral tumor cell lines between 30 and 50%, compared with the non-treated controls. However, cell-line-specific effects are known to occur and natural compounds and extracts, such as CE and GSE used in this study, may produce their effects either directly or indirectly [33] via multiple pathways, including apoptosis-related pathways, such as caspase-2, caspase-3, and -8 [13, 34–36]. A further analysis of these specific molecular pathways in CAL27 and SCC25 cells, under CE and GSE administration, revealed a common, dramatic up-regulation of mRNA expression in both the apoptosis initiator, caspase-2, and the apoptosis effector, caspase-8, from CAL27 and SCC25 cell lines within 24 h; unambiguous indicators that these specific apoptosis-inducing factors were directly affected by these treatments and thus may be involved, to some degree, in the observed phenotypic changes.

Although some phenotypic changes, such as proliferation inhibition, were comparable, other phenotypic changes and molecular pathways altered by CE and GSE were strikingly dissimilar. CE reduced cellular adhesion in both cell lines, while GSE increased SCC25 adhesion and concomitantly increased cell-cell clustering, but produced no significant effects in CAL27 cells. This differs profoundly from our previous study using purified GSE-derived PAC, which produced no observable changes to either cell clustering or cell-cell adhesion [18]. Analysis of molecular pathways, commonly associated with oral tumor proliferation, revealed further dissimilarities, most notably that GSE reduced mRNA expression of p53, c-myc and ODC in SCC25 cells, while CE enhanced their expression in the CAL27 cell line. Because damaged genes and molecular pathways can vary from tumor to tumor, genetic heterogeneity may offer one potential explanation of how these tumor cell lines might behave differently under the same treatment—suggesting this study may represent a crucial first step toward explaining which components may be involved with the CE- or GSE-mediated phenotypic changes in oral cancer proliferation.

Both CE and GSE contain multiple substances, in addition to their monomeric and oligomeric PACs, which may have competing effects on cellular phenotypes despite their relatively low concentrations [1]. Carotenoids, dithioltiones, glucosinolates, indoles, isothiocyanates, protease inhibitors, plant sterols, allium compounds, limonenes, selenium, vitamin C, vitamin E and dietary fiber, which may be present in both CE and GSE, may be responsible for modulating and mediating a variety of cellular phenotypes and may be involved in the differing phenotypic alterations described in this study [1, 4]. In addition, these substances are likely to influence multiple pathways in addition to those that are directly or indirectly modulated by PACs [34]. For example, although PACs, GSE and other flavonoid extracts are known to activate apoptosis-related pathways [13, 35–37], while inhibiting aromatase [38] and ornithine decarboxylase (ODC) in epithelial tumors [9, 39, 40], these additional trace substances found in CE and GSE may exhibit differing, or even competing, effects which must also be considered potential confounders.

Because botanical extracts may have additive or synergistic effects, as well as competing or confounding effects [33], comparisons of these effects on well-characterized tumor cell lines provide a framework and model for the discussion and analysis of potential biological mechanisms that produce clinical effects [41, 42]. Such studies have helped to identify chemopreventive and chemotherapeutic agents that provide selective effects against cancerous cells without untoward effects to normal cells and tissues [43]. The growing evidence that CE and GSE may be effective adjuvants and complementary treatments for breast, colon and prostate cancers, combined with these results, suggests the possibility that CE and GSE may provide selective inhibition of oral cancers, at least *in vitro*. Because evidence now suggests that CE, GSE or PACs, administered as dietary supplements, are non-toxic and bioavailable in both serum and tissues at *μ*g/ml concentrations, they may be promising candidates for further exploration as adjuvant or complementary therapies for patients with, or at risk of developing, oral cancers [44, 45].

## Supplementary Data

Supplementary data are available at eCAM Online.

## Supplementary Material

CE administration significantly inhibited CAL27 proliferation in vitro, GSE administration inhibited CAL27 proliferation in vitro, CE administration inhibited SCC25 proliferation in vitro, GSE administration significantly inhibited SCC25 proliferation in vitro, CE inhibited adhesion of OSCC cell lines in vitro while GSE had variable effects, effects of CE and GSE on CAL27 cell morphology in vitro, effects of CE and GSE on SCC25 cell morphology in vitro, effects of CE and GSE on cell morphology in 30-min adhesion assays, and proliferation inhibition of CAL27 comparison: PAC, CE, and GSE were all presented in the figures.

## Figures and Tables

**Table 1:** CE- and GSE-induced effects on CAL27 and SCC25 adhesion.

	[0] *μ*g/mL	[10] *μ*g/mL	[20] *μ*g/mL	[30] *μ*g/mL	[40] *μ*g/mL	[50] *μ*g/mL	[60] *μ*g/mL	[70] *μ*g/mL	[80] *μ*g/mL
**CAL27: CE**									
AVE	0.169708	0.158833	0.140667	0.133083	0.136417	0.125792	0.132958	0.1185	0.126
STD	0.071633	0.046028	0.045456	0.03347	0.029181	0.031508	0.038637	0.024	0.027107
% change		−0.064081	−0.171127	−0.215811	−0.19617	−0.258777	−0.216548	**−0.301743**	−0.25755
**CAL27: GSE**									
AVE	0.189542	0.190417	0.163875	0.168625	0.186417	0.172917	0.1755	0.171708	0.177625
STD	0.064007	0.058357	0.039513	0.04395	0.068084	0.069489	0.060548	0.05999	0.052694
% change		0.004616	**−0.135414**	−0.110354	−0.016487	−0.087712	−0.074082	−0.094087	−0.062871
**SCC25: CE**									
AVE	0.154542	0.146792	0.145958	0.150333	0.137042	0.124583	0.129625	0.141875	0.158875
STD	0.046158	0.029719	0.02906	0.044132	0.051973	0.028853	0.051599	0.046965	0.078825
% change		−0.050148	−0.055541	−0.027231	−0.113238	**−0.193853**	−0.161229	−0.081963	0.02804
**SCC25: GSE**									
AVE	0.16625	0.164625	0.16675	0.191708	0.176833	0.193208	0.198083	0.195333	0.227167
STD	0.052803	0.035287	0.034081	0.054233	0.049174	0.117639	0.071255	0.06047	0.081278
% change		−0.009774	0.003008	0.153133	0.063659	0.162155	0.191479	0.174937	**0.366416**

Total *n* = 216; sample per assay, *n* = 24.
