# Supplementary material for: Cranberry and Grape Seed Extracts Inhibit the Proliferative Phenotype of Oral Squamous Cell Carcinomas
Source: Evid Based Complement Alternat Med. 2010 Oct 18;2011:467691. doi: 10.1093/ecam/nen047 (PMC3138501; doi:10.1093/ecam/nen047)
Supplement: Supplementary file 1 — CE administration significantly inhibited CAL27 proliferation in vitro, GSE administration inhibited CAL27 proliferation in vitro, CE administration inhibited SCC25 proliferation in vitro, GSE administration significantly inhibited SCC25 proliferation in vitro, CE inhibited adhesion of OSCC cell lines in vitro while GSE had variable effects, effects of CE and GSE on CAL27 cell morphology in vitro, effects of CE and GSE on SCC25 cell morphology in vitro, effects of CE and GSE on cell morphology in 30-min adhesion assays, and proliferation inhibition of CAL27 comparison: PAC, CE, and GSE were all presented in the figures. [file 467691.f1.pdf]

A

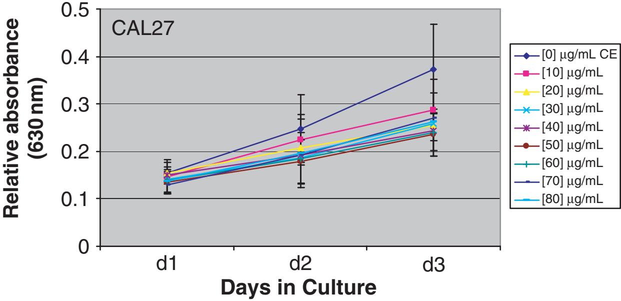

B

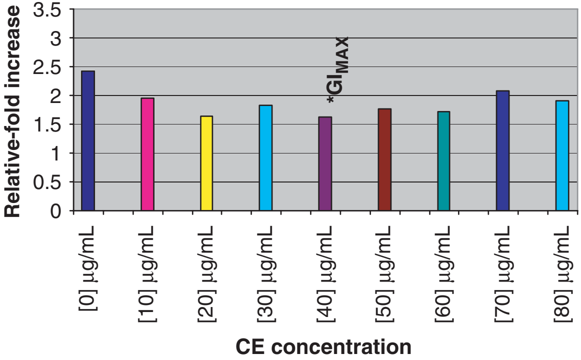

C

ANOVA

	Sum of Squares	df	Mean Square	F	Sig.
Between	.456	8	.029	5.999	.000
Within	1.284	279	.005		
Total	1.740	287			

two-tailed *t*-test (p value)

	+CE [10 μg/mL]	+CE [40 μg/mL]
CAL27	0.005893	0.000000
