# Supplementary material for: Cranberry and Grape Seed Extracts Inhibit the Proliferative Phenotype of Oral Squamous Cell Carcinomas
Source: Evid Based Complement Alternat Med. 2010 Oct 18;2011:467691. doi: 10.1093/ecam/nen047 (PMC3138501; doi:10.1093/ecam/nen047)
Supplement: Supplementary file 2 [file 467691.f2.pdf]

A

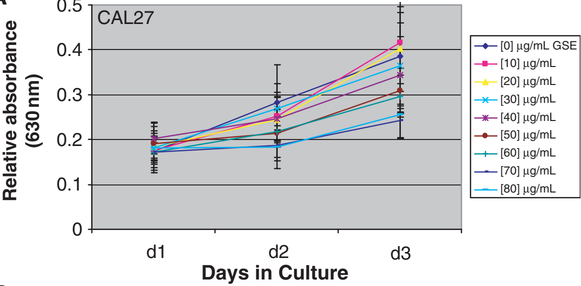

B

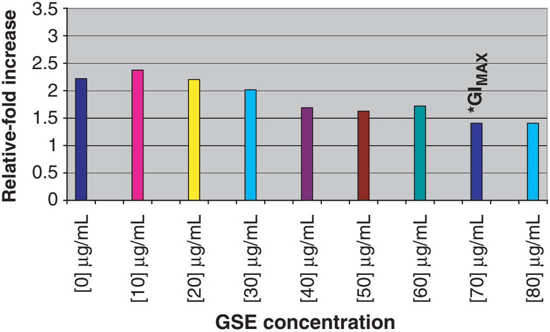

C

ANOVA

	Sum of Squares	df	Mean Square	F	Sig.
Between	.515	8	.064	8.552	.000
Within	2.036	279	.008		
Total	3.064	287			

two-tailed *t*-test (p value)

	+GSE [10 µg/mL]	+GSE [70 µg/mL]
CAL27	0.5269	0.000000
