# Supplementary material for: Cranberry and Grape Seed Extracts Inhibit the Proliferative Phenotype of Oral Squamous Cell Carcinomas
Source: Evid Based Complement Alternat Med. 2010 Oct 18;2011:467691. doi: 10.1093/ecam/nen047 (PMC3138501; doi:10.1093/ecam/nen047)
Supplement: Supplementary file 3 [file 467691.f3.pdf]

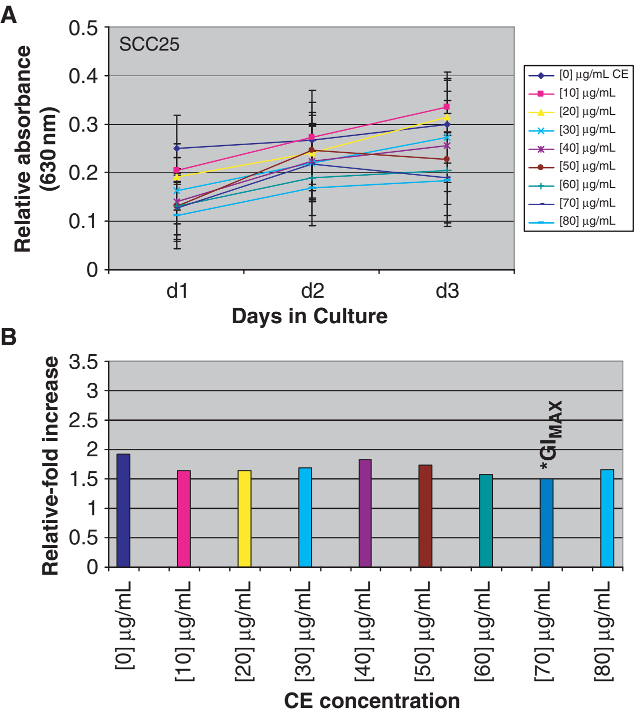

C

ANOVA

	Sum of Squares	df	Mean Square	F	Sig.
Between	.663	8	.083	6.706	.000
Within	3.450	279	.012		
Total	4.114	287			

two-tailed *t*-test (p value)

	+CE [10 µg/mL]	+CE [70 µg/mL]
SCC25	0.462246	0.000353
