# Supplementary material for: Cranberry and Grape Seed Extracts Inhibit the Proliferative Phenotype of Oral Squamous Cell Carcinomas
Source: Evid Based Complement Alternat Med. 2010 Oct 18;2011:467691. doi: 10.1093/ecam/nen047 (PMC3138501; doi:10.1093/ecam/nen047)
Supplement: Supplementary file 4 [file 467691.f4.pdf]

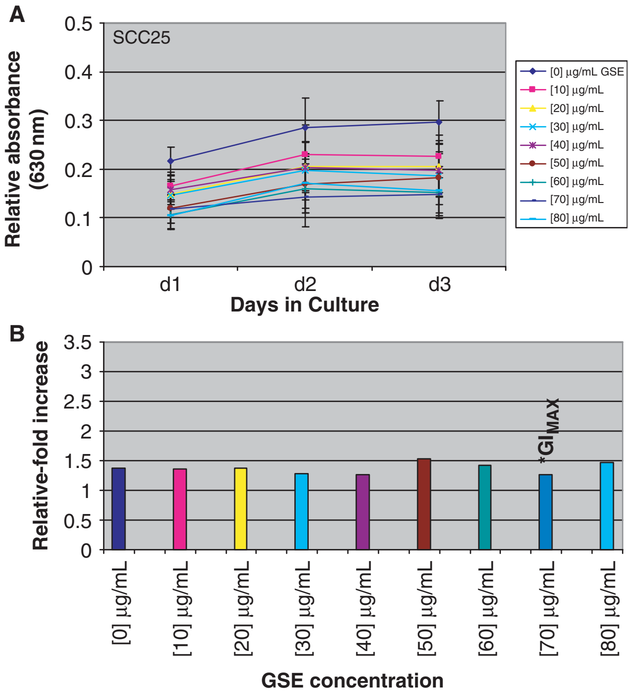

C

ANOVA

	Sum of Squares	df	Mean Square	F	Sig.
Between	.573	8	.072	14.542	.000
Within	1.375	279	.005		
Total	1.949	287			

two-tailed *t*-test (p value)

	+GSE [10 µg/mL]	+GSE [70 µg/mL]
SCC25	0.005894	0.000451
