# Supplementary material for: Cranberry and Grape Seed Extracts Inhibit the Proliferative Phenotype of Oral Squamous Cell Carcinomas
Source: Evid Based Complement Alternat Med. 2010 Oct 18;2011:467691. doi: 10.1093/ecam/nen047 (PMC3138501; doi:10.1093/ecam/nen047)
Supplement: Supplementary file 5 [file 467691.f5.pdf]

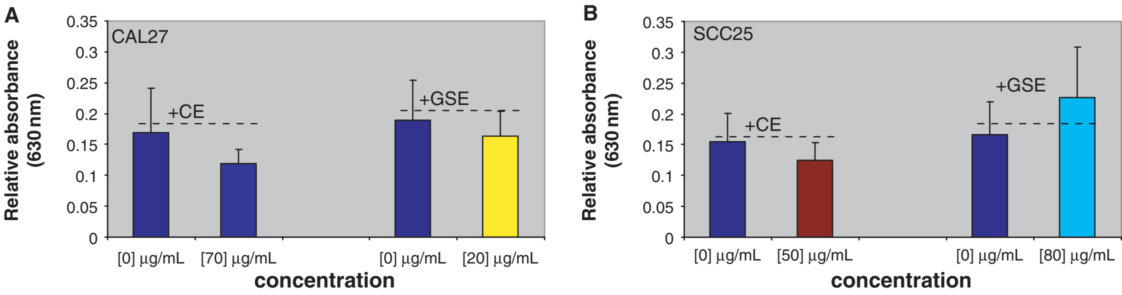

two-tailed *t*-test (p value)

	+CE [70 μg/mL]	+GSE [20 μg/mL]
CAL27	0.0024	0.1027*

two-tailed *t*-test (p value)

	+CE [50 μg/mL]	+GSE [80 μg/mL]
SCC25	0.010	0.003
