# Supplementary figures and images for: Cranberry and Grape Seed Extracts Inhibit the Proliferative Phenotype of Oral Squamous Cell Carcinomas
Source: Evid Based Complement Alternat Med. 2010 Oct 18;2011:467691. doi: 10.1093/ecam/nen047 (PMC3138501; doi:10.1093/ecam/nen047)

CAL27

CAL27

+CE

+GSE

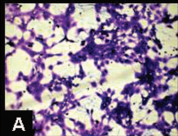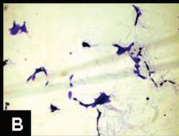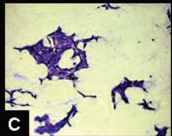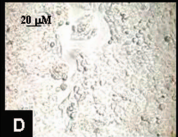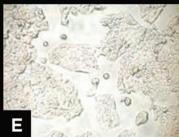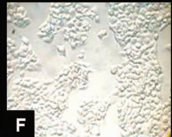

Supplement: Supplementary file 6 [file 467691.f6.pdf]

**+CE**

**+GSE**

**SCC25**

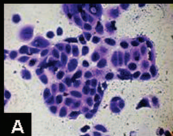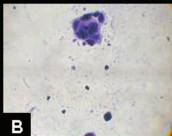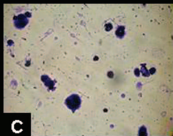

**SCC25**

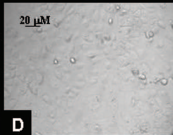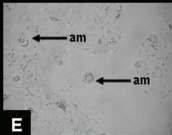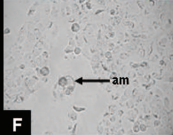

Supplement: Supplementary file 7 [file 467691.f7.pdf]

CAL27

SCC-25

+CE

+GSE

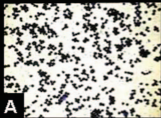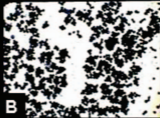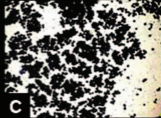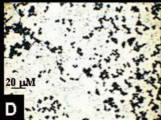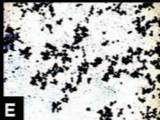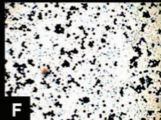

Supplement: Supplementary file 8 [file 467691.f8.pdf]
