# Supplementary material for: Cranberry and Grape Seed Extracts Inhibit the Proliferative Phenotype of Oral Squamous Cell Carcinomas
Source: Evid Based Complement Alternat Med. 2010 Oct 18;2011:467691. doi: 10.1093/ecam/nen047 (PMC3138501; doi:10.1093/ecam/nen047)
Supplement: Supplementary file 9 [file 467691.f9.pdf]

A

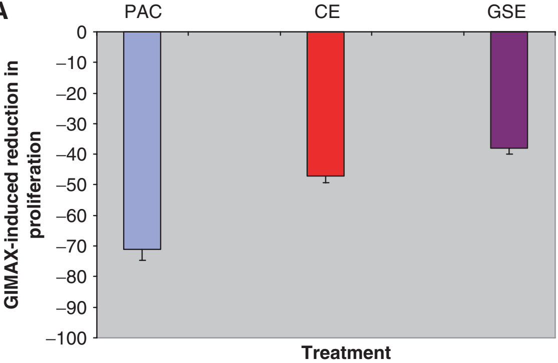

B

	Control	PAC 50μg/mL GI_{MAX}	CE 40μg/mL GI_{MAX}	GSE 70μg/mL GI_{MAX}
CAL27 Cell viability (% change)	82%	10% (–72%)	80% (–2%)	70% (–11%)
CAL27 Cell spreading (% change)	85%	5% (–80%)	75% (–10%)	70% (–15%)
CAL27 Confluence (% change)	45%	15% (–67%)	30% (–34%)	28% (–38%)
